# Supplementary material for: Causal interplay between lactose intolerance and gut microbiota: a combined bidirectional Mendelian randomization and in vivo validation study
Source: Front Nutr. 2026 Jun 1;13:1803337. doi: 10.3389/fnut.2026.1803337 (PMC13265576; doi:10.3389/fnut.2026.1803337)
Supplement: Supplementary file 1 [file Data_Sheet_1.zip › supplementary materials/Forward/leave one out cross validation/ebi-a-GCST90027851.finngen_R12_E4_LACTONAS.pdf]

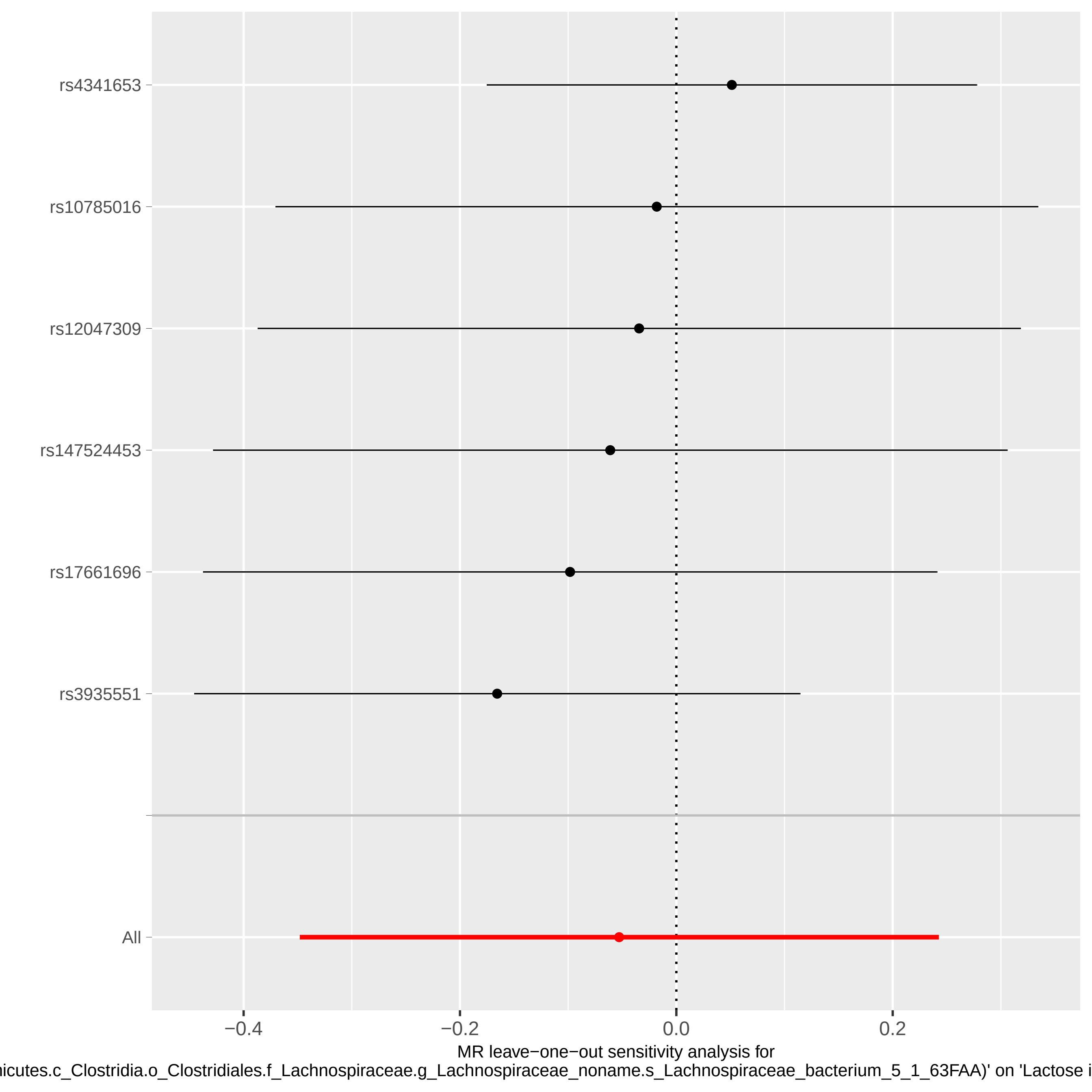

rs4341653
rs10785016
rs12047309
rs147524453
rs17661696
rs3935551
All
−0.4
−0.2
0.0
0.2
MR leave−one−out sensitivity analysis for
nicutes.c_Clostridia.o_Clostridiales.f_Lachnospiraceae.g_Lachnospiraceae_noname.s_Lachnospiraceae_bacterium_5_1_63FAA)' on 'Lactose i
